# Multiparametric dynamic contrast-enhanced ultrasound imaging of prostate cancer

**DOI:** 10.1007/s00330-016-4693-8

**Published:** 2016-12-21

**Authors:** Rogier R. Wildeboer, Arnoud W. Postema, Libertario Demi, Maarten P. J. Kuenen, Hessel Wijkstra, Massimo Mischi

**Affiliations:** 1 Laboratory of Biomedical Diagnostics, Department of Electrical Engineering, Eindhoven University of Technology, PO-Box 513, 5600 MB Eindhoven, The Netherlands; 2 Department of Urology, Academic Medical Center University Hospital, Meibergdreef 9, 1105 AZ Amsterdam, The Netherlands; 3 Philips Research, Philips, Eindhoven, The Netherlands

**Keywords:** Prostate cancer, Ultrasound, Contrast agents, Classification, Multiparametric imaging

## Abstract

**Objectives:**

The aim of this study is to improve the accuracy of dynamic contrast-enhanced ultrasound (DCE-US) for prostate cancer (PCa) localization by means of a multiparametric approach.

**Materials and Methods:**

Thirteen different parameters related to either perfusion or dispersion were extracted pixel-by-pixel from 45 DCE-US recordings in 19 patients referred for radical prostatectomy. Multiparametric maps were retrospectively produced using a Gaussian mixture model algorithm. These were subsequently evaluated on their pixel-wise performance in classifying 43 benign and 42 malignant histopathologically confirmed regions of interest, using a prostate-based leave-one-out procedure.

**Results:**

The combination of the spatiotemporal correlation (*r*), mean transit time (*μ*), curve skewness (*κ*), and peak time (PT) yielded an accuracy of 81% ± 11%, which was higher than the best performing single parameters: *r* (73%), *μ* (72%), and wash-in time (72%). The negative predictive value increased to 83% ± 16% from 70%, 69% and 67%, respectively. Pixel inclusion based on the confidence level boosted these measures to 90% with half of the pixels excluded, but without disregarding any prostate or region.

**Conclusions:**

Our results suggest multiparametric DCE-US analysis might be a useful diagnostic tool for PCa, possibly supporting future targeting of biopsies or therapy. Application in other types of cancer can also be foreseen.

***Key points*:**

• *DCE*-*US can be used to extract both perfusion and dispersion*-*related parameters*.

• *Multiparametric DCE*-*US performs better in detecting PCa than single*-*parametric DCE*-*US*.

• *Multiparametric DCE*-*US might become a useful tool for PCa localization*.

## Introduction

Prostate cancer (PCa) is the most prevalent form of cancer among American men, representing 26% of the new cases and approximately 10% of the cancer-related deaths [1]. Therefore, a reliable and minimally invasive diagnostic tool for PCa is of paramount importance. During the last decade of the 20th century, the introduction of prostate-specific antigen (PSA) blood testing led to a dramatic increase of the number of PCa diagnoses as well as a growing number of patients exhibiting low-risk or indolent disease [2]. Overdiagnosis and overtreatment are considered substantial problems due to the limited positive predictive value (PPV) of screening tools such as PSA level assessment and digital rectal examination [3, 4]. Therefore, the definitive diagnosis of PCa still relies on ≥10 core systematic biopsy [5]. There is a high incidence of biopsy-related complications [6, 7] and a considerable fraction of malignancies is identified only in repeat biopsy [8]. This stresses the demand for an imaging modality that is able to localize or rule out prostatic malignancies. Such a technique could eventually serve as a localization tool for targeted biopsy [9, 10], or assist in patient selection and treatment planning for organ-sparing focal therapy [11].

Currently, multiparametric magnetic resonance imaging (mpMRI) seems the most promising imaging method for PCa localization [12]. A recent meta-analysis reported an appreciable average sensitivity of 74% and specificity of 88%, with negative predictive values (NPVs) that ranged from 65% to 94% [13]. In view of the advantages of transrectal ultrasound (TRUS) over MRI in terms of costs, time, resolution, and practicality at bedside, this paper proposes a multiparametric approach of TRUS.

Unfortunately, B-mode TRUS is not considered sufficiently accurate for stand-alone tumour detection [14, 15]. Since clinically relevant prostatic malignancies are characterized by angiogenesis and neovascularization [16, 17], increased perfusion has been proposed as a marker for PCa. Moreover, it was observed that the microvascular density correlates with cancer aggressiveness [18]. However, Doppler imaging was not found sufficiently accurate to capture these vascular changes due to its limited sensitivity for small flows [19, 20]. The use of intravenously injected ultrasound contrast agents (UCAs) in the TRUS procedure, that is, dynamic contrast-enhanced ultrasound (DCE-US) imaging, also allows the visualization of the vascular fraction and perfusion. Contrast-specific imaging modes are even able to image microcirculation at a capillary scale [21]. Again, despite the reported improvements in tumour detection rate [22, 23], targeted biopsies based on visual interpretation of contrast-enhanced US alone are not considered viable to replace systematic biopsy [9, 24]. This might be explained by the inconsistent, ambiguous effect of angiogenesis on blood flow [25]. Whereas diminished vasomotor control and formation of shunts cause an elevation in perfusion, the high tortuosity and permeability, with rising interstitial pressure, lead to the opposite effect [16, 17].

Tissue characterization by DCE-US thus requires a more detailed assessment of the UCA kinetics in the prostate. UCAs are composed of encapsulated micron-sized gas bubbles that remain a few minutes in the vasculature [26–28], and their behaviour can be assessed by looking at the time-intensity curve (TIC), which is the evolution of echo intensity over time in a certain area. For example, malignant areas in DCE-US recordings are found to be marked by rapid and enhanced inflow compared to similar benign regions in the prostate [29, 30]. Several parameters have been extracted from the TICs in order to mark relevant alterations in perfusion; these are, e.g., the wash-in rate [31, 32], time to peak (PT) [33, 34], time to appearance (AT) [33, 34], peak intensity (PI) [32–35], and the area under the curve [33, 36]. In addition to these perfusion-related parameters, angiogenic microvasculature changes can also be detected by assessing UCA dispersion [37]. Contrast US dispersion imaging (CUDI) was recently developed to analyse the dispersion kinetics of a microbubble contrast bolus in a DCE-US recording. The dispersive behaviour has been assessed with curve fitting [37], or similarity analysis [38, 39]. In these methods, the TIC of each pixel in the imaging plane is either fitted by a convective diffusion model or compared to its neighbouring TICs, respectively.

Though different in nature, the parameters acquired with these methods were shown to have appreciable levels of sensitivity and specificity for PCa detection. Hence, we hypothesize that a combination of complementary parameters related to perfusion and dispersion allows us to localize prostatic carcinoma with an even higher level of accuracy. PCa is a multifocal and heterogeneous disease whose appearance depends on cancer type, grade and topography [24]. A multiparametric approach reduces the risk of missing tumours that are invisible to one of the parameters and may be able to discriminate prostatic diseases that mimic malignant characteristics, like prostatitis [40].

Since the performance of a multiparametric approach is not dependent on a single threshold, it cannot be evaluated using conventional receiver operating characteristic (ROC) analysis [41, 42]. Instead, a multiparametric approach requires a classification algorithm to combine the parameters into a single parametric map. Many algorithms have been used in biomedicine for classification; in particular, Gaussian mixture models (GMMs), support vector machines and artificial neural networks have been extensively employed [43–45]. GMMs have been chosen for our multiparametric evaluation as these are fast, purely based on data (no need for additional physical modelling) and facilitate the definition of classification confidence. Moreover, GMMs were reported to perform better than neural networks in mammographic tumour identification [45].

In this paper, we take into account thirteen perfusion- and dispersion-related parameters as well as the echo intensity on the TRUS image. Retrospectively, the most useful parameters are selected and combined using histopathologically determined regions of interest (ROIs) in order to improve the accuracy of DCE-US for the localization of PCa.

## Materials and methods

### Data acquisition

Nineteen PCa patients that were scheduled for radical prostatectomy underwent a transrectal DCE-US scan prior to surgery. The procedures were approved by the local ethics committee and carried out at the Academic Medical Center (Amsterdam, The Netherlands). All patients signed an informed consent. Patients below the age of 18 or with contraindications for the administration of contrast agents as defined by the European Medicines Agency were excluded. Patients with a tumour of Gleason score ≥ 3 + 3 and a size suitable for our analysis (see section “Histopathological Analysis”) were selected for this study. The patient and tumour characteristics are summarized in Table 1.

**Table 1:** List of patient and tumour characteristics

Characteristic	Mean	Median	Range
Age (yrs)	62.7	64	52 – 73
PSA level (ng/mL)	8.7	7.3	2.9 – 31.9
Prostate volume (mL)	35.8	30	20 – 83.5
	Number		
Clinical stage			
T1	0		
T2	11		
T3	8		
Gleason score			
3 + 3	6		
3 + 4	7		
4 + 3	4		
3 + 5	1		
4 + 5	1		

For the procedure, 2.4 mL of a SonoVue® UCA microbubble suspension (Bracco, Milan, Italy) was intravenously administered. This suspension consists of encapsulated sulphur hexafluoride bubbles with an average diameter of 2.5 μm [46]. Two-minute recordings were subsequently performed with an iU22 US scanner (Philips Healthcare, Bothell, WA, USA), generally using a 3-MHz to 10-MHz-ranged endocavity US probe (C10-3v). For one patient, an endocavity probe with a range from 4 MHz to 8 MHz (C8-4v) was used due to availability issues. The measurements were carried out in contrast-specific mode based on a power modulation pulse scheme at a frequency of 3.5 MHz and with a mechanical index of 0.06 to minimize bubble disruption.

### Histopathological analysis

After prostatectomy, the prostates were fixed with a formalin solution, deprived of the seminal vesicles, sectioned in slices of ~4 mm and examined by the pathologist as described by Montironi et al. [47]. The tumours were subsequently delineated by the pathologist. Prior to the parametric analysis, ~0.5 cm^2^-sized ROIs were manually drawn on the B-mode US scans to identify areas containing histologically confirmed malignancy. Histological images were matched to the US scans based on the position of the imaging plane in a transversal sweep video performed before the contrast recordings. ROIs were only drawn in areas where the histopathologic information persisted in the two adjacent slices. The prostatic boundary was used to aid the localization. In the same way, we positioned similarly sized ROIs in areas that were not depicted as malignant. This resulted in a dataset containing about 174,000 pixels extracted from 85 ROIs in 45 DCE-US recordings in 19 patients, marked as either benign (43 ROIs) or malignant (42 ROIs). In the end, we were able to include one to four DCE-US imaging planes per patient.

### Data processing and parameter extraction

To assess the dispersive UCA behaviour, the DCE-US recordings were analysed by TIC fitting as well as by similarity analysis. The parametric maps were produced using a custom-made CUDI program running in Matlab® (2015b, Mathworks, Natick, MA, USA) [48]. Following the method described in [37], the data were pre-processed and the extracted TICs were fitted by a modified local density random walk (LDRW) model. This allowed us to estimate the area under the curve (*α*), the mean transit time (*μ*), the skewness parameter (*κ*), and the ratio between the diffusive and convective time (*λ* = *μκ*). In addition, we looked at the variance (var) which is the second moment of the curve [49], and the fitting interval (int) between the PT and the truncation time where UCA recirculation occurs [37].

For the similarity analysis, the data were pre-processed as described in [39]. The spectral coherence (*ρ*) [38] and the spatiotemporal correlation coefficient (*r*) [39] were then calculated. For these parameters, the TIC of a single pixel is compared to those in a ring-shaped kernel of 1.0 to 2.5 mm in radius, as this size allows us to visualize similarity on the scale of early angiogenesis [38].

Based on the processing in [37], we also extracted the PI, the AT (where the TIC reaches 5% of the PI) and the PT (the time where the intensity is the highest). In addition, the wash-in time (WIT, the time period between AT and the point where the TIC reaches 95% of the PI), and the full width half maximum (FWHM) were considered as parameters of interest. A full list of the investigated parameters is reported in Table 2.

**Table 2:** Full list of the parameters considered for multiparametric analysis, with symbols and units

Symbol	Parameter name	Unit
B-mode ultrasound		
Greylevel	Echo intensity	a.u.
Contrast-enhanced ultrasound		
WIT	Wash-in time	s
AT	Appearance time	s
PT	Peak time	s
PI	Peak intensity	a.u.
FWHM	Full width half maximum	s
Fitting analysis		
*κ*	Skewness parameter	s^-1^
*μ*	Mean transit time	s
*λ*	Convective-diffusion ratio	-
*α*	Area under the curve	a.u.
var	Variance	a.u.
int	Interval time	s
Similarity analysis		
*ρ*	Spectral coherence	-
*r*	Correlation coefficient	-

### Classification procedure

Gaussian mixture modelling is a widely known approach in cluster analysis [50, 51] and data classification [52, 53]. GMMs describe a set of observations (i.e. pixels) in (multi)parametric space by a mixture of normal distributions. Using two pre-determined training subsets of benign and malignant observations, the class-specific probability distributions can be computed. Subsequently, each pixel in the test set is classified according to these distributions. We define a measure for the confidence of classification by comparing the probabilities, *p*, of the observation being benign or malignant. This confidence level, *P*, conveniently ranging from 0 to 1, is described by $P=\frac{2{p}_A}{p_A+{p}_B}-1$, where *A* denotes the class with highest probability.

The GMM classification algorithm was implemented in Matlab® using the statistical analysis toolbox. The parameters were normalized to the 90th percentile to ensure equal weighting, and training was performed using an iterative expectation-maximization algorithm [54]. Since the GMM algorithm is very fast, it was feasible to evaluate all possible combinations of one to four distinct parameters. We did not take into account more than four parameters to avoid overfitting.

To evaluate the performance of the classifier, the procedure was tested on each of the prostates whilst using the observations in other prostates as the training set. The outcomes of this leave-one-out analysis were averaged over all prostates. We quantified the classification performance by computing the accuracy, sensitivity, specificity, PPV and NPV [41, 42]. Whereas sensitivity and specificity indicate the percentage of correctly classified malignant and benign pixels, respectively, PPV and NPV reflect the percentage of pixels classified respectively as malignant and benign that were correct. In addition, accuracy represents the overall correct classification percentage. Since the NPV is of paramount importance in order to avoid missing clinically relevant PCa lesions, we optimized the classifier based on this measure as well as on the accuracy.

### Pixel exclusion

The TIC measurement quality usually differs from pixel to pixel. To ensure the quality of the classification, it is important to identify pixels that are likely to be misclassified. As stated, the classification algorithm indicates the confidence of the classification by the level *P*. Also, we consider the coefficient of determination, *R* ^2^, describing how well the TIC can be fitted by the modified LDRW model, and the absolute probability of an observation belonging to its class. Large healthy vessels are more likely to show early arrival of the bolus, which complicates the use of perfusion parameters to mark malignancy. Therefore, we also evaluated the classifier’s performance after excluding the pixels with the lowest PT for each plane.

## Results

Based on accuracy, the combination of *r*, *μ*, *κ*, and PT yielded the best accuracy (mean ± standard deviation = 81 ± 11%). The highest NPV was found for the parameters var, *μ*, *r*, and int (87 ± 15%), but with two of the other performance measures being inferior compared to first set. The high NPV and sensitivity can be explained by a low number of false negatives. We found the parameter distributions best described by a single Gaussian function per variable. The outcomes were compared to the performance of individual parameters. The best-performing parameters of all three analyses—*μ* for curve fitting, *r* for similarity analysis and WIT for conventional perfusion analysis—were evaluated by a ROC-based threshold optimization as well as GMM classification in one-dimensional parametric space. As shown in Table 3, the multiparametric classification has a higher performance than the ROC-analysed single parameters, irrespective of the measure used. Since not all individual parameters are well described by a single Gaussian distribution, a non-tailored GMM approach for the single parameters yielded less stable results in terms of the balance between sensitivity and specificity. Even though accuracy and NPV are considered the most important performance measures, a reliable technique requires the other measures to be sufficiently high as well.

**Table 3:** Performance of the classification methods using specified parameters

	ROC Analysis			Single GMM			Multiparametric GMM	
	WIT	*μ*	*r*	WIT	*μ*	*r*	*r*, *μ*, *κ*, PT	var, *μ*, *r*, int
Accuracy (%)	72	72	73	73	71	67	81	72
Sensitivity (%)	75	74	71	88	90	65	79	90
Specificity (%)	68	70	75	51	47	71	80	50
PPV (%)	76	75	76	70	63	70	85	65
NPV (%)	67	69	70	84	85	72	83	87

The used abbreviations are listed in the Abbreviations List and Table 2

The best performing perfusion-related parameter as well as best parameters of curve fitting and similarity analysis were evaluated using ROC analysis and single-parameter GMM. Multiparametric results are shown of the parameter sets with the highest accuracy (*r*, *μ*, *κ,* PT) and NPV (var, *μ*, *r*, int)

**Fig. 1:**
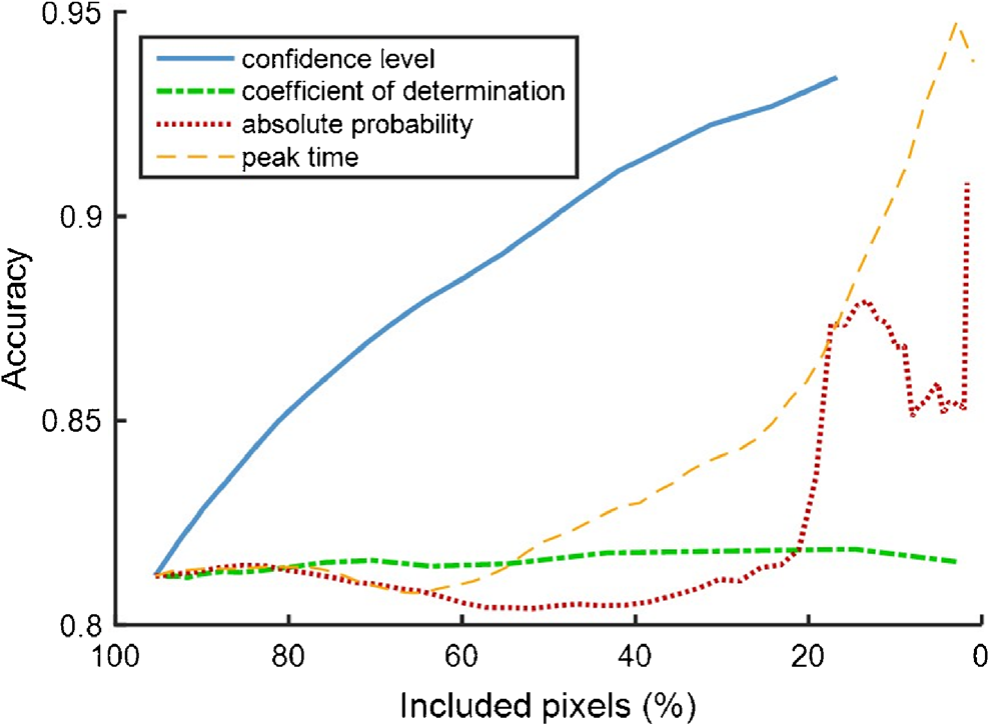
Accuracy of the Gaussian mixture model classifier by exclusion of pixels based upon their confidence level, coefficient of determination, absolute probability, and peak time. The classifier was run using a set of parameters containing *r*, *μ*, *κ*, and PT

**Fig. 2:**
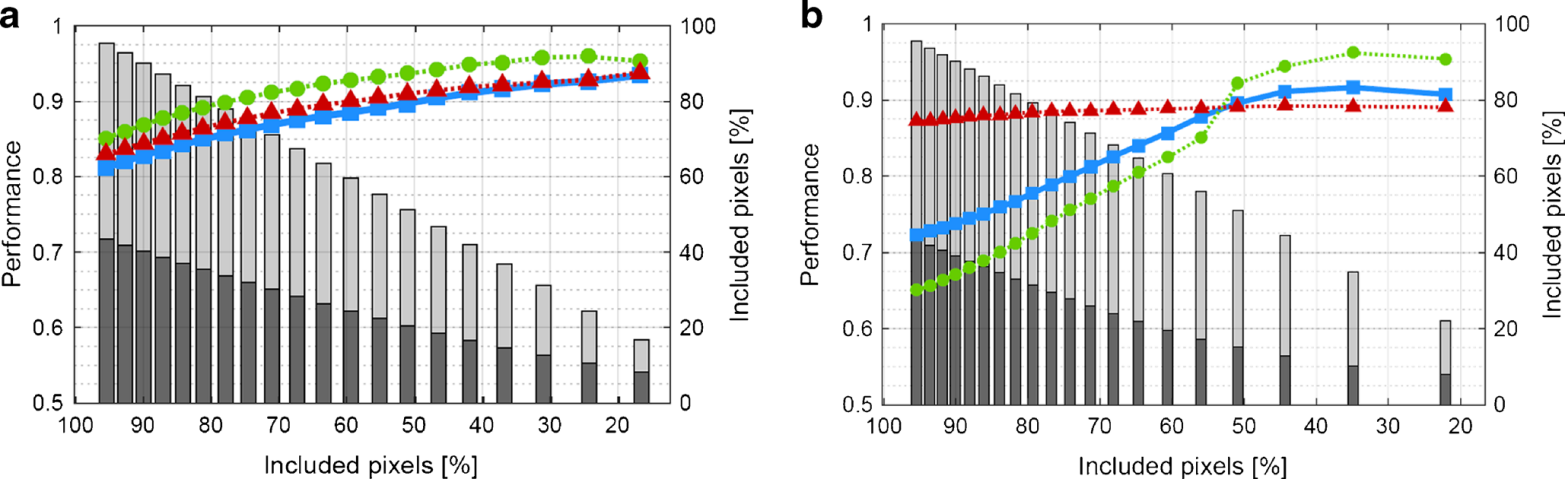
Performance of the Gaussian mixture model classifier upon exclusion of pixels with the lowest confidence using an increasing confidence threshold for (a) *r*, *μ*, *κ*, PT and (b) var, *μ*, int, *r*. The bars represent the percentage of benign (grey) and malignant (dark) pixels that are still included; the lines represent the evolution of accuracy (blue squares ■), NPV (red triangles ▲), and PPV (green circles ●)

In the “Materials and methods” section, we mentioned feature-based exclusion of pixels to decrease the number of misclassifications in the multiparametric map. Figure 1 shows the changes in accuracy after pixel exclusion based on classification confidence *P*, absolute probability, *R* ^2^, and PT. It reveals that *P* correctly reflects the confidence and that it is the most suitable measure to identify pixels with a high risk of misclassification. The results of the multiparametric classification after pixel exclusion based on this measure are depicted in Figures 2a and b for a parameter set containing *r*, *μ*, *κ*, and *PT* and a set containing var, *μ*, *r*, and int, respectively. The exclusion of pixels is equally distributed over benign and malignant pixels, as well as over patients and regions; exclusion would, therefore, not result in extra PCa foci being missed.

**Fig. 3:**
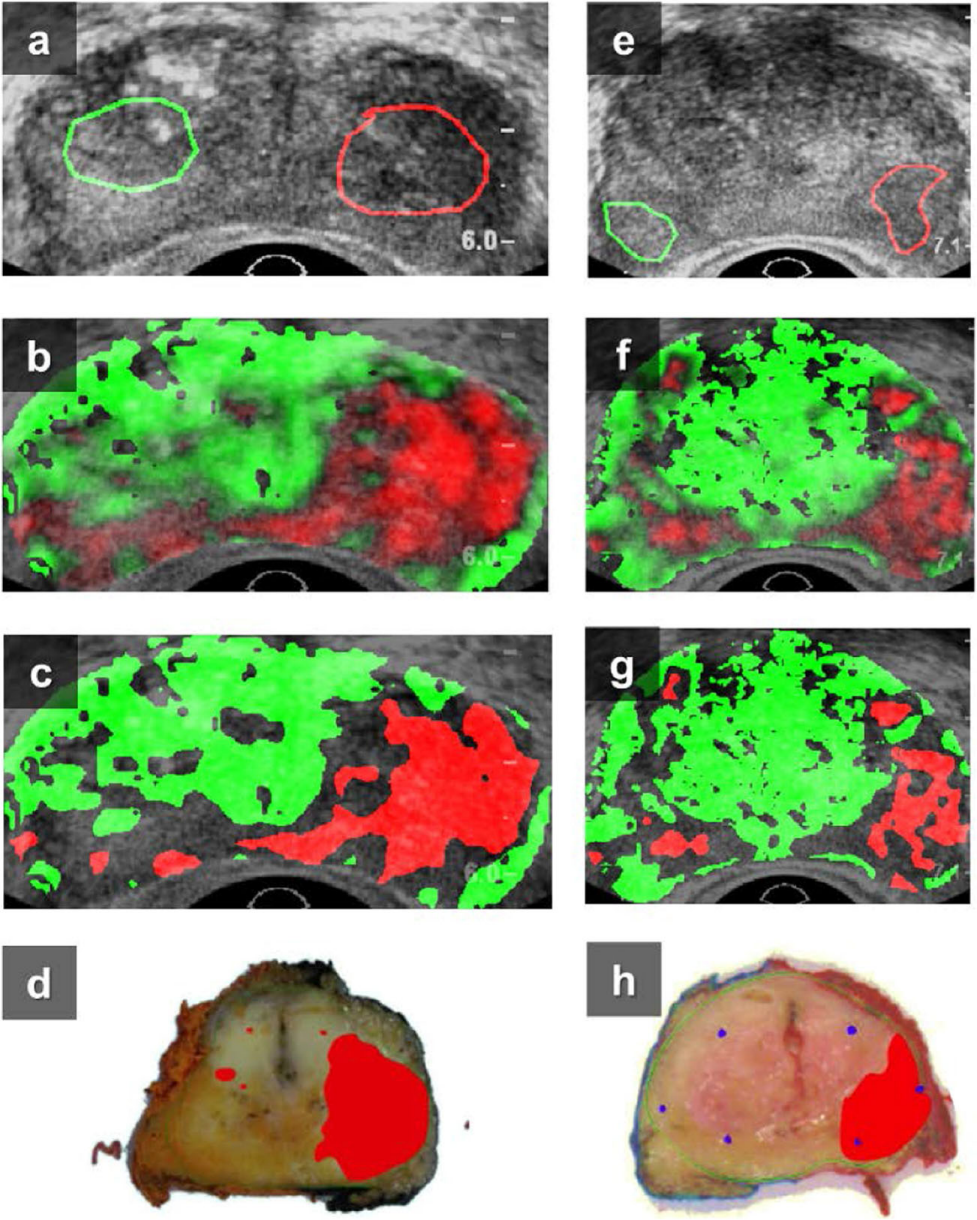
The B-mode transrectal ultrasound, confidence-weighted classification image, exclusion-classification images with a threshold of *P* > 0.5 and histopathological images of patient A (a t/m d) and patient B, (e t/m h). In the classification images, red regions are classified as malignant (i.e. suspicious) and green regions as benign (i.e. not suspicious). In the histopathological images, malignant areas are indicated with red. Parameters: *r*, *μ*, *κ*, and PT. ROIs are shown in overlay to the B-mode images

**Fig. 4:**
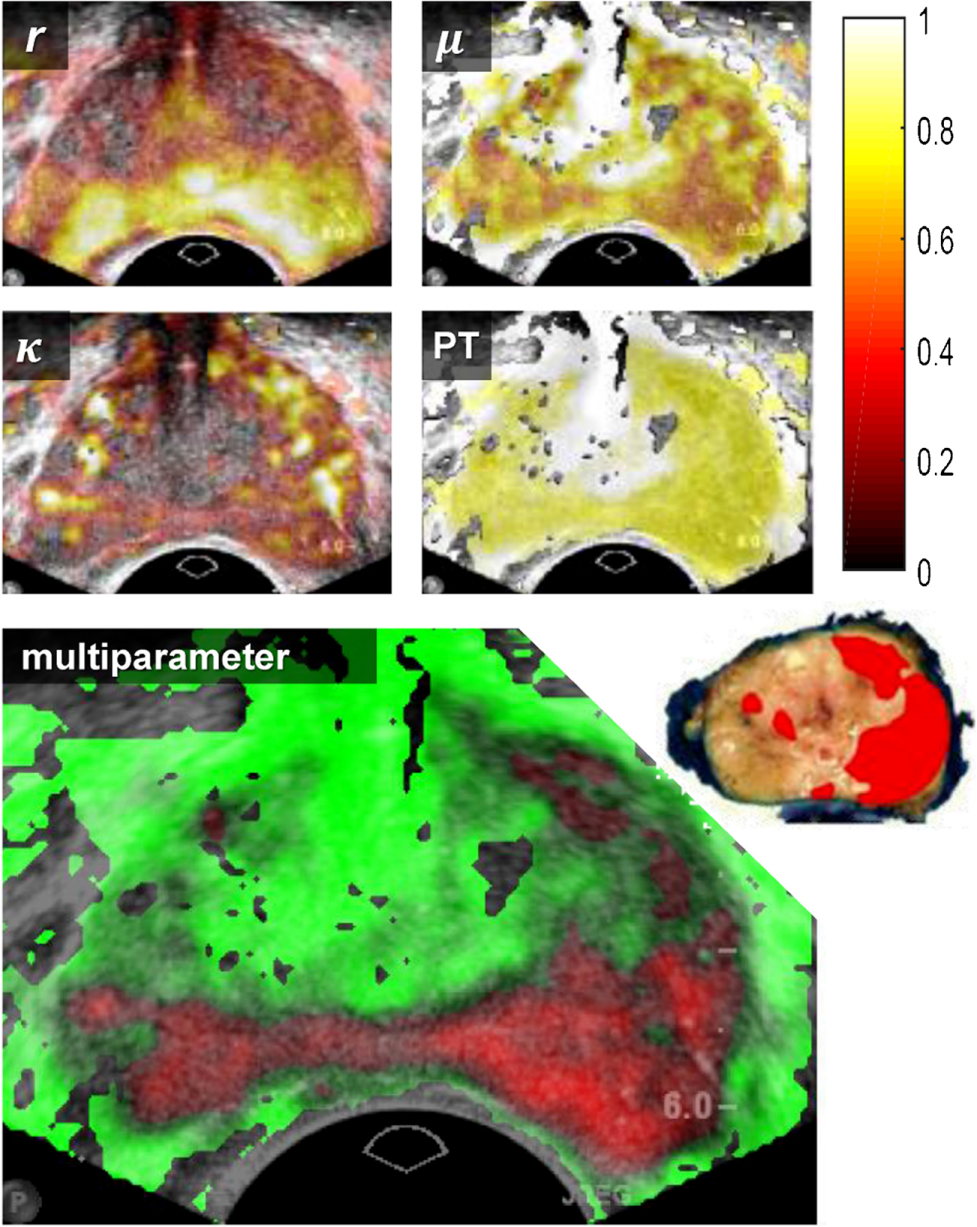
Example of the four normalized parametric maps that serve as input for the best performing multiparametric map as shown below. All maps overlay the B-mode TRUS image. Red regions are classified as malignant (i.e. suspicious) and green regions as benign (i.e. not suspicious) of which the transparency is scaled with the confidence level. The histology slice with tumour tissue marked red is shown in the upper right corner of the multiparametric image

To illustrate the results of whole-prostate classification and the effect of pixel exclusion, the US and classification maps as well as histological images of two patients are shown in Figure 3. For the first set in Figure 2, the accuracy and NPV have grown from 81 ± 11% to 90 ± 10% and from 83 ± 16% to 91 ± 13% with 51 ± 17% of the pixels remaining. These values are 72 ± 10% to 90 ± 7% and 87 ± 15% to 89 ± 15% for the second set, again with 51 ± 14% of the pixels included. These exclusion percentages include the 4.5 ± 3.2% of pixels that could not be fitted by the LDRW model. For reference, Figure 4 depicts the individual, normalized parametric maps that contribute to a multiparametric image.

## Discussion

According to today’s guidelines [5], reliable PCa diagnosis requires a ≥10 core systematic biopsy under US guidance and local anaesthesia. In recent years, an increasing emphasis has been laid on imaging and targeted biopsy [9] in view of the number of reported complications [6], over-diagnoses due to the overestimation of pathologically insignificant lesions [3], and under-diagnoses due to small high-risk PCa foci being missed [8]. Contrast-enhanced US allows the extraction of multiple parameters that have potential to serve as a diagnostic marker for malignancy. The presented multiparametric approach combines perfusion-related parameters from conventional DCE-US and dispersion-related parameters from CUDI by means of a GMM classifier.

The optimal subset of parameters comprises *r*, *μ*, *κ*, and PT and thus features parameters from all analysis methods. Of these parameters, *r* contributes most to the outcome, which is consistent with previous publications on CUDI [39]. As *μ* and *κ* jointly describe the shape of LDRW-modelled TIC [55], it is not surprising the combination of these two has the greatest added value to *r*. Finally, the addition of PT offers a slight improvement to the accuracy (80% to 81%). Though early enhancement is a strong marker of malignancy, the quantitative use of the PT is normally complicated by its strong dependence on operator and circulation time [34]. In combination with the other parameters, however, the PT is able to further delineate malignant and benign regions. Despite its good performance as a single parameter, the WIT is not included in the multiparametric sets. We expect that this is the result of the high correlation between WIT and *μ* (Pearson’s *r*: 0.88), making this parameter redundant after inclusion of *μ*.

We have shown that observations that are likely to be misclassified can be recognized by their low confidence level. Excluding low-confidence pixels from the multiparametric map increases the reliability of the classification. A confidence threshold of 0.5 leads to an average pixel exclusion of 36 ± 15% per prostate (ranging from 8 to 67%). In general, the pixel exclusion approach resulted in disregarding pixels in the areas where benign regions border on malignant ones rather than pixels in specific prostates. As can be seen in Figure 2, exclusion does not favour malignant or benign pixels specifically. Figure 3 shows typical examples of classification maps. There is a high correspondence between the maps and histology, even though small regions remain misclassified after pixel exclusion. Due to the preservation of the correctly classified malignant regions, these results are clinically relevant for, e.g. targeted biopsy. Because the current analysis faces limitations with respect to the registration of US imaging planes with histology slices, we made use of histologically proven ROIs. These ROIs were ~0.5-cm^2^ sized, which resembles the critical size of clinically relevant foci [47]. In the future, three-dimensional US models would enable us to apply more accurate registration.

In the current analysis, 9 (20%) of the 44 regions predominantly (>50% of the pixels) classified as negative were misclassified. In Table 1, Gleason score 3 + 3 represents most of these false negative regions. The Gleason score, which comprises the grade of the two most prevalent histological patterns found in a stained prostate tissue slice, is an indicator of the stage and aggressiveness of prostatic carcinoma [56, 57]. Following recent consensus in prostate grading, Gleason rate score 3 + 3 is rated as grade group 1, indicating very low-risk disease with high survival rates and virtually no chance of metastasis [58, 59]. We believe that the use of a diverse training set (i.e. a set that consists of malignancies with varying aggressiveness) leads to a higher risk of misclassification in prostates containing very low-grade or very high-grade PCa, as these are the most different from the training set average.

The current performance is limited by the small training set in this study, hampering the possibility to define subgroups according to Gleason score. Since the microvascular density is a viable marker in the staging of PCa [18], this might allow us to distinguish low-risk and high-risk PCa. Studies in contrast-enhanced MRI suggest that perfusion-based discrimination between PCa grades, and even prostatitis, is possible [60]. In this study, we did not assess the differences in tumour classification of grades; an extended dataset would allow such validation in the future. Another limitation of the study concerns the analysis of small foci, which, considering the error margin in appointing the ROIs, could not be included in the study.

DCE-US is not only a valuable modality for diagnosis of PCa; its use is increasingly mentioned as a tool to monitor the therapeutic effect of focal therapy. For instance, DCE-US was found to map tissue devascularisation as well as DCE-MRI after interstitial laser therapy [61, 62]. DCE-US has, therefore, been used for interstitial laser therapy [63] and high-intensity focused US treatment to visualize viable and devascularized regions [64, 65]. Apart from the classifier’s aid for tumour localization and monitoring, the ability of such classifier to discriminate low-risk and high-risk disease would especially improve the application of focal therapy and active surveillance strategies.

In conclusion, we see that combined evaluation of contrast-enhanced ultrasonographic parameters has superior accuracy and NPV in tumour localization compared to the individual parametric maps. The GMM-based multiparametric analysis is fast, versatile and allows a reliable confidence estimation of its classification. It was shown that pixel exclusion could boost the performance even more without disregarding relevant areas of the prostate. Like in computer-aided diagnosis of breast lesions [66, 67], an extensive review of other algorithms is recommended to obtain an overview of the performance, advantages and drawbacks of other classification methods for the detection of PCa. In the future, parametric maps derived from other US modalities such as Doppler and elastography could also be included [68], as well as the results from other diagnostic tools (e.g. PSA assessment), but this is beyond the scope of the current study. Furthermore, this analysis is based on a small patient group, and we recognize that a more extended validation is needed to derive global measures for classification. We expect that this method can also be employed to image other types of cancer.

## Abbreviations

AT: Appearance time
CUDI: Contrast-ultrasound dispersion imaging
DCE-US: Dynamic contrast-enhanced ultrasound
FWHM: Full width half mximum
GMM: Gaussian mixture model
LDRW: Local density random walk
mpMRI: Multiparametric magnetic resonance imaging
NPV: Negative predictive value
PCa: Prostate cancer
PPV: Positive predictive value
PSA: Prostate-specific antigen
PT: Peak time
ROC: Receiver operating characteristic
ROI: Region of interest
TIC: Time-intensity curve
TRUS: Transrectal ultrasound
UCA: Ultrasound contrast agent
WIT: Wash-in time
